# Combined influence of depression and low-grade inflammation on mortality in peritoneal dialysis patients

**DOI:** 10.1186/s12882-023-03291-2

**Published:** 2023-08-17

**Authors:** Yanxia Zhang, Jiexin Chen, Ruiying Tang, Jihong Deng, Huankai Guo, Xianfeng Wu, Qingdong Xu

**Affiliations:** 1 Department of Nephrology, Jiangmen Central Hospital, Jiangmen, China; 2 Department of Nephrology, Affiliated Sixth People’s Hospital, Shanghai Jiao Tong University, Shanghai, China

**Keywords:** Depression, Low-grade inflammation, Peritoneal dialysis, Mortality

## Abstract

**Background:**

The relationship between depression and systemic inflammation as risk factors for mortality is not well understood and requires further investigation.

**Methods:**

Patients undergoing continuous ambulatory peritoneal dialysis (CAPD) between July 01, 2015 to December 31, 2019, were analyzed and followed up until December 31, 2020. According to their status of depression (PHQ-9 score ≥ 5) and low-grade inflammation (hs-CRP level ≥ 3 mg/L), patients were divided into four groups (G1, without depression, nor inflammation; G2, with depression, without inflammation; G3, with inflammation, without depression; G4, with both depression and inflammation). We performed Kaplan–Meier and multivariable Cox proportional analyses of mortality for the combined influence of depression and systemic inflammation in this cohort.

**Results:**

During the mean follow-up of 36.3 ± 14.8 months, 73 deaths were recorded in 358 participants. Compared with patients in group G1, patients in group G2 and G3 carried 137% {hazard ratio (HR): 2.37, 95% confidence interval (CI): 1.06—5.23, *p* = 0.035} and 140% (HR: 2.40, 95% CI: 1.01—5.69, *p* = 0.048) higher risk of mortality. Patients in group G4 (with both depression and inflammation) showed the highest risks of all-cause mortality with 276% higher mortality risk (HR: 3.76, 95% CI: 1.73—8.15, *p* = 0.001), respectively.

**Conclusion:**

The combined of depression and inflammation is associated with all-cause mortality in peritoneal dialysis patients, suggesting a need for further study of depression and low-grade inflammation in PD patients and potential relationship between them.

## Introduction

In the world's population, the prevalence of chronic kidney disease (CKD) is estimated to be 9.1%, with 0.041% of patients being treated by dialysis [1]. Moreover, the global all-age CKD mortality rate has increased by 41·5% from 1990 to 2017. Among dialysis patients, approximately 11% of them receive peritoneal dialysis (PD) [2]. Although PD is an efficient therapy for renal replacement, the mortality rate in PD patients is still high. In addition to traditional risk factors such as age, diabetes [3], primary combined cardiovascular disease (CVD) [4], high body mass index (BMI) [5], peritonitis [6] and high peritoneal transport status [7], there is increasing interest in the relationship between psychosocial risk factors and poor outcomes of PD patients, including depression, anxiety and lower social support [8].

Depression is one of the most prevalent psychiatric disorders, with a prevalence of 17% in the general population [9]. Furthermore, the prevalence of depression was respectively higher in the continuous ambulatory peritoneal dialysis (CAPD) group compared to the normal estimated glomerular filtration rate (eGFR) and CKD stages 1–2 group [10, 11]. The diagnosis of depression is independently associated with death or hospitalization of patients on chronic hemodialysis [12]. Inflammation has been shown to play an important role in the progression and mortality of various diseases. Exposed to glucose-based peritoneal dialysis fluid, patients undergoing PD have a higher risk of hyperglycemia, which is associated with oxidative stress and inflammation. Indeed, high levels of pro-inflammatory cytokines like interleukin-6 (IL-6), tumor necrosis factor-α [13] and interleukin-1β (IL-1β) [14], may promote the onset of depression, as well as inflammatory markers like C-reactive protein (CRP) [15]. Given the bidirectional mechanism between depression and inflammation, the combined influence of these two factors on mortality in PD patients is unknown so far.

What’s more, most depression patients are easily ignored and inadequately treated in the past decade. Previous studies only assessed the relationship of depression and PD patients at the baseline [16]. In addition, published evidence that examined depression levels impacting clinical outcome including mortality risk, has only included patients on hemodialysis [12]. This study explored the combined influence of depression and inflammation on mortality in PD patients and highlights the importance of identifying controllable factors and improved management of risk factors for PD patients.

## Methods

### Study patients

In this cohort study, patients who received continuous ambulatory peritoneal dialysis (CAPD) from July 01, 2015 to December 31, 2019, were enrolled and followed-up. The exclusion criteria were as follows: patients aged < 18 years at the start of PD; those who received PD therapy for less than 3 months; those who were not able to answer the questionnaires reliably; those with acute infection complications (such as peritonitis, pulmonary infection, sepsis and diarrhea) and those with malignant tumors. Finally, 358 participants were included in the study and followed up until endpoint or December 31, 2020. This study was approved by the Human Ethics Committees of study organization.

### Clinical variables

Baseline demographic data included gender, age, etiology of ESRD, history of hypertension, diabetes, CVD and medications use. Clinical and biochemical data included BMI, hypersensitivity C-Reactive Protein (hs-CRP), hemoglobin, serum albumin, serum calcium, serum phosphorus, serum intact parathyroid hormone (iPTH), total cholesterol (TC) and triglyceride (TG). Serum CRP was analyzed by immunoturbidimetry. High sensitivity plasma CRP level was dichotomized into two categories: 0–3.0 mg/L was defined as normal and ≥ 3 mg/L is considered a clinically significant status of low-grade inflammation [17]. All baseline data were obtained during the first 3 months of PD.

### Psychometric assessment of depression

Depressive symptoms were assessed using the Patient Health Questionnaire (PHQ-9) for screening and classification of depression [18]. PHQ-9 consists of 9 questions corresponding to the 9 criteria for defining depression according to Diagnostic and Statistical Manual Fourth Edition (DSM-IV) [18]. Each question was scored from 0 point (i.e. not at all) to 3 points (i.e. nearly every day) according to severity. Overall score was computed and patients were classified according to their severity of depressive symptoms, from none, mild, moderate, moderately severe, and severe (with PHQ-9 score of 0 to 4, 5 to 9, 10 to 14, 15 to 19, and ≥ 20, respectively).

Patients were divided into four groups based on the status of depression and status of low-grade inflammation: group 1 (G1, without depression, nor inflammation); group 2 (G2, with depression, without inflammation); group 3 (G3, with inflammation, without depression); group 4 (G4, with both depression and inflammation).

### Follow-up and endpoint

The primary outcomes included all-cause mortality. All patients were followed up until death, cessation of PD (receiving renal transplantation, transferring to hemodialysis), loss of follow-up, or the end of follow-up (December 31, 2020).

### Statistical analyses

Data are expressed as the mean ± standard deviation (SD), percentages, or median (25 – 75% interquartile range). Continuous variables were compared using analysis of variance or the Kruskal–Wallis test, and categorical variables were tested using the *χ*^2^ test or Fisher’s exact test. Kaplan–Meier curve was used to compare survival between different groups. Univariate and multivariate Cox proportional hazards regression models were applied to identify independent prognostic factors of outcomes. The results are presented as the hazard ratios (HR) and reported with 95% confidence intervals (CI). Statistical analyses were conducted using Statistical Package Social Science Vision 26.0 (IBM SPSS 26.0). *P* values < 0.05 were considered significant.

## Results

### Baseline characteristics and correlations between different statuses of depression and inflammation with clinical parameters

A total of 358 patients were enrolled in this study. Among them, the average age was 47.3 ± 15.3 years and 59.2% were female. According to the PHQ-9 score, 147 patients (41.1%) were classified as not depressed, 211 patients (58.9%) were depressed, with.

95 (26.5%) mildly depressed, 72 (20.1%) moderately depressed, 26 (7.3%) moderately severe depressed, and 18 (5.0%) severely depressed. 147 (41.1%) were with low-grade inflammation. Compared with the reference group (G1: *n* = 85, without depression, nor inflammation), patients in group2 (*n* = 126, with depression, without inflammation), group3 (*n* = 62, with inflammation, without depression) and group4 (*n* = 85, with both depression and inflammation) tended to be older and had higher level of phosphorus (Table 1, all* p* < 0.05).

**Table 1:** Baseline characteristics of individuals stratified by depression severity and low-grade inflammation

Variables	Total	Group 1	Group 2	Group 3	Group 4	*p* value
	(*n* = 358)	(*n* = 85)	(*n* = 126)	(*n* = 62)	(*n* = 85)	
**Sociodemographic factors**						
Gender, female (%)	212 (59.2)	49 (57.6)	74 (58.7)	35 (56.5)	54 (63.5)	0.813
Age (years)	47.3 ± 14.0	43.8 ± 13.5	45.1 ± 13.3	51.2 ± 14.9	51.1 ± 13.6	< 0.001
BMI (kg/m^2^)	21.8 ± 3.3	21.0 ± 3.4	22.0 ± 3.2	21.9 ± 3.4	22.2 ± 3.2	0.114
Etiology of ESRD						0.386
Chronic glomerulonephritis (%)	229 (64.0)	63 (74.1)	81 (64.3)	35 (56.5)	50 (58.8)	
Diabetic nephropathy (%)	75 (20.9)	15 (17.6)	24 (19.0)	15 (24.2)	21 (24.7)	
Hypertensive nephropathy (%)	26 (7.3)	4 (4.7)	10 (7.9)	4 (6.5)	8 (9.4)	
Others (%)	28 (7.8)	3 (3.5)	11 (8.7)	8 (12.9)	6 (7.1)	
**Comorbid conditions**						
Hypertension (%)	318 (88.8)	74 (87.1)	113 (89.7)	56 (90.3)	75 (88.2)	0.912
Diabetes (%)	91 (25.4)	17 (20)	28 (22.2)	17 (27.4)	29 (34.1)	0.137
History of CVD (%)	190 (53.1)	44 (51.8)	72 (57.1)	31 (50.0)	43 (50.6)	0.719
**Medication**						
ACE-inhibitor (%)	42 (12.2)	6 (7.6)	19 (15.8)	4 (6.6)	13 (15.5)	0.127
Angiotensin receptor blocker (%)	159 (46.2)	37 (46.8)	55 (45.8)	30 (49.2)	37 (44.0)	0.942
Calcium-antagonists (%)	295 (85.8)	68 (86.1)	106 (88.3)	49 (80.3)	72 (85.7)	0.546
Beta-blocker (%)	211 (61.3)	46 (58.2)	75 (62.5)	37 (60.7)	53 (63.1)	0.916
**Laboratory variables**						
PHQ-9 score	7.4 ± 6.1	2.0 ± 1.4	11.0 ± 5.3	2.1 ± 1.4	11.4 ± 5.0	< 0.001
hs-CRP (mg/L)	4.1 ± 4.1	1.1 ± 0.8	1.4 ± 0.9	7.2 ± 3.3	8.6 ± 3.9	< 0.001
Hemoglobin (g/L)	106.4 ± 14.0	106.9 ± 14.2	106.0 ± 14.2	106.7 ± 14.8	106.2 ± 13.2	0.965
Albumin (g/L)	35.6 ± 4.9	36.1 ± 4.6	36.2 ± 4.4	34.9 ± 5.0	34.7 ± 5.9	0.077
Calcium (mmol/L)	2.2 ± 0.2	2.3 ± 0.2	2.2 ± 0.2	2.3 ± 0.2	2.2 ± 0.2	0.351
Phosphorus (mmol/L)	1.8 ± 0.6	1.7 ± 0.5	1.8 ± 0.5	1.8 ± 0.6	2.0 ± 0.7	0.022
Intact Parathyroid hormone (pg/mL)	524.7 ± 436.1	493.0 ± 393.8	487.2 ± 411.8	514.1 ± 428.2	619.8 ± 505.4	0.141
Cholesterol (mmol/L)	4.9 ± 1.3	5.0 ± 1.6	4.8 ± 1.1	5.0 ± 1.2	4.8 ± 1.3	0.636
Triglyceride (mmol/L)	1.8 ± 1.3	1.8 ± 1.4	1.6 ± 1.0	2.0 ± 1.5	2.0 ± 1.5	0.057
Total Kt/V	2.4 ± 0.7	2.4 ± 0.7	2.4 ± 0.7	2.5 ± 0.7	2.3 ± 0.7	0.572
eGFR (mL/min/1.73 m^2^)	5.7 ± 4.3	5.5 ± 4.2	5.8 ± 4.2	5.3 ± 3.7	5.9 ± 5.1	0.805

*Abbreviations*: *BMI* body mass index, *ESRD* end-stage renal disease, *CVD* cardiovascular disease, *ACE* angiotensin-converting enzyme, *PHQ* Patient Health Questionnaire, *total Kt/V* the sum of peritoneal and renal Kt/Vurea, *eGFR* estimated glomerular

Spearman’s analyses revealed that severity of depression was correlated with hs-CRP (*r* = 0.133), combined hypertension (*r* = 0.105) and the level of phosphorus (*r* = 0.120) (all *p* < 0.05, Table 2). While inflammation level was correlated with depression (*r* = 0.133), age (*r* = 0.256), etiology of ESRD (*r* = 0.125), combined hypertension (*r* = 0.143), phosphorus (*r* = 0.134), iPTH (*r* = 0.113) and triglyceride (*r* = 0.156), but negatively correlated with albumin (*r* = -0.138) (all *p* < 0.05, Table 3). Tables 2 and 3 revealed that most parameters have very weak correlation with depression as well as inflammation (-0.2 < 0.2), except age which has correlation with inflammation level (*r* = 0.256).

**Table 2:** Spearman correlation analysis between depression severity and clinical parameters

	r	*p* value
hs-CRP	0.133^*^	0.012
Gender, female (%)	-0.001	0.979
Age (years)	-0.035	0.506
BMI (kg/m^2^)	0.037	0.481
Etiology of ESRD	0.086	0.103
Hypertension	0.105^*^	0.047
Diabetes	-0.011	0.832
History of CVD	-0.010	0.855
Hemoglobin (g/L)	-0.023	0.660
Albumin (g/L)	0.004	0.944
Calcium (mmol/L)	-0.068	0.201
Phosphorus (mmol/L)	0.120^*^	0.023
Intact Parathyroid hormone (pg/mL)	0.052	0.331
Cholesterol (mmol/L)	-0.043	0.415
Triglyceride (mmol/L)	-0.044	0.408
Total Kt/V	0.027	0.607
eGFR (mL/min/1.73 m^2^)	0.027	0.617

*Abbreviations*: *BMI* body mass index, *ESRD* end-stage renal disease, *CVD* cardiovascular disease, *total Kt/V* the sum of peritoneal and renal Kt/Vurea, *eGFR* estimated glomerular.

*:*p* < 0.05

**Table 3:** Spearman correlation analysis between inflammation level and clinical parameters

	r	*p* value
depression	0.133^*^	0.012
Gender, female (%)	0.021	0.696
Age (years)	0.256^**^	0.000
BMI (kg/m^2^)	0.067	0.206
Etiology of ESRD	0.125^*^	0.018
Hypertension	0.143^**^	0.007
Diabetes	-0.041	0.438
History of CVD	-0.008	0.882
Hemoglobin (g/L)	0.019	0.720
Albumin (g/L)	-0.138**	0.009
Calcium (mmol/L)	-0.053	0.316
Phosphorus (mmol/L)	0.134^*^	0.011
Intact Parathyroid hormone (pg/mL)	0.113^*^	0.033
Cholesterol (mmol/L)	-0.018	0.738
Triglyceride (mmol/L)	0.156^**^	0.003
Total Kt/V	-0.002	0.972
eGFR (mL/min/1.73 m^2^)	0.012	0.821

*Abbreviations*: *BMI* body mass index, *ESRD* end-stage renal disease, *CVD* cardiovascular disease, *total Kt/V* the sum of peritoneal and renal Kt/Vurea, *eGFR* estimated glomerular.

*:*p* < 0.05, **:*p* < 0.01

### Effect of combined influence of depression and inflammation on all-cause mortality in patients undergoing PD

**Fig. 1:**
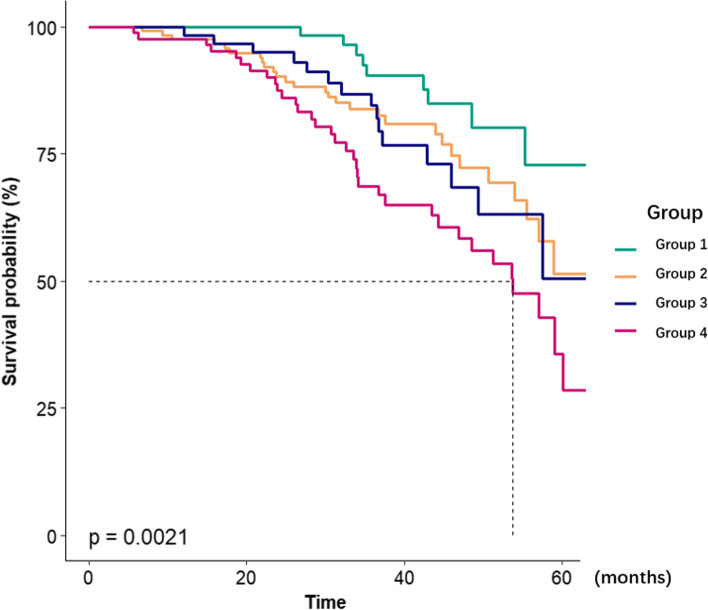
Effect of combined influence of depression and inflammation on all-cause mortality in patients undergoing PD

During the mean follow-up of 36.3 ± 14.8 months, 73 participants died. The Kaplan–Meier curves indicated that compared with the reference group (G1: without depression, nor inflammation), patients in G4 (with both depression and inflammation) had a shorter overall survival for all-cause mortality (Fig. 1, *p* = 0.002).

### Independent prognostic factors for all-cause mortality in patients undergoing PD

The effect of the combined depression and inflammation on all-cause mortality was demonstrated using Cox proportional hazard regression models. Univariate analysis revealed that status with both depression and inflammation was a risk factor for all-cause mortality. After fully adjusting for gender, age, BMI, Etiology of ESRD, history of hypertension, diabetes, CVD, HB, Alb, calcium, phosphorus, iPTH, TC, TG, total Kt/V and eGFR, multivariable analysis revealed that patients in group G2 and G3 carried 137% (HR: 2.37, 95% CI:1.06—5.23, *p* = 0.035) and 140% (HR: 2.40, 95% CI:1.01—5.69, *p* = 0.048) higher risk of mortality. It is worth noting that, patients in group G4 (with both depression and inflammation) showed the highest risks of all-cause mortality with 276% higher mortality risk (HR:3.76, 95% CI:1.73—8.15, *p* = 0.001), respectively (Table 4).

**Table 4:** The associations of stratification (depression severity and low-grade inflammation) with all-cause mortality

	n(%)	Model 1:(unadjusted)		Model 2	
		HR	*p* value	HR	*p* value
Group 1	9 (10.59%)	ref		ref	
Group 2	28 (22.22%)	2.13 (1—4.51)	0.049	2.37 (1.06—5.28)	0.035
Group 3	15 (24.19%)	2.20 (0.96—5.04)	0.061	2.40 (1.01—5.69)	0.048
Group 4	34 (40%)	3.67 (1.76—7.66)	0.001	3.76 (1.73—8.15)	0.001

Model 2: adjusted for gender, age, BMI, etiology of ESRD, hypertension, diabetes, history of CVD, hemoglobin, albumin, calcium, phosphorus, intact parathyroid hormone, cholesterol, triglyceride, total Kt/V and eGFR

## Discussion

Our study shows that depression and low-grade Inflammation are common in PD patients. Notely, individuals with both depression and inflammation exhibited the highest risks of all-cause mortality. To our knowledge, this is the first study focusing on PD patients to evaluate the combined influence of depression and inflammation in predicting mortality, which may provide an additional information on mortality risk in this population.

Depression is one of the most prevalent psychiatric disorders in ESRD patients. The totally prevalence of depression in our study was 58.9% (with 26.5% mildly, 20.1% moderately, 7.3% moderately, 5.0% severely depressed) which is higher than other studies focus on dialysis patients (43%-55%) [19, 20]. This discrepancy might be explained by differences in population characteristics (HD or PD, incident or prevelant), study design, sample size, and the measurement tool used to evaluate depression [21].

In previous studies, uncontrolled hypertension [22] leads to anxiety, and PD patients are advised to maintain dietary restrictions to avoid hyperphosphatemia, which might result in depression due to the burden of dietary restrict and poor quality of life [23]. In addition to traditional risk factors, there is growing interest in the possible interaction between somatic and psychiatric symptoms in PD patients. As a result, depression has been confirmed to be associated with tumor, infection, and especially the development of CVD [24, 25]. The potential mechanism of poor outcome includes hypothalamic–pituitary–adrenal gland dysfunction, increased proinflammatory and prothrombotic factor activity, reduced heart rate variability and slight physical inactivity [26]. Moreover, relationships between major depressive disorder and other medical conditions were confirmed in previous studies, including chronic kidney disease-mineral and bone disorder (CKD-MBD), lipid metabolism disorder and malnutrition/protein-energy wasting (PEW) [27, 28]. However, most clinical parameters showed weak correlation with inflammation and depression in this study. This might mean that combination of these parameters contributes to and depression rather than merely just a single factor in predominant effect.

Approximately 41.1% of PD patients in this study are considered to have clinically significant status of low-grade inflammation. Furthermore, our findings revealed that higher levels of inflammatory markers (hs-CRP) were associated with more severe depressive symptoms. The mechanisms underlying the correlation between inflammation and depression among PD patients are not yet completely understood. Nonetheless, chronic inflammation may contribute to the development of depression through several pathways. For example, inflammation can activate microglia immune cells in the central nervous system which play a role in regulating mood and behavior [29]. Additionally, previous study demonstrated that patients with major depression exhibited a reduction in TNF-αand CRP levels following treatment with antidepressants [30].

In this study, multivariate Cox analysis revealed that the co-occurrence of depression and inflammation in patients was independently linked to the greatest risks of all-cause mortality. The exact biological mechanisms linking depression and increased mortality risk remain unclear. However, studies exploring patients without known renal disease suggest that inflammation-related atherosclerotic cardiovascular diseases may be responsible for depression-induced mortality [31]. Two different models were used to examine the potential impact of depression and inflammation on mortality in previous studies. On one hand, moderation analysis showed that an interaction effect of depression by inflammation was not significantly associated with mortality, suggesting that the mortality risk conferred by increased levels of inflammation is not further augmented by depression [32]. In line with another comprehensive analysis, it showed that inflammation played a significant additive-but not interactive-effects with depression in mortality risk [18]. On the other hand, the mediation effect of CRP (mediator) accounted for 7.3% of the relationship between depression and all-cause mortality [33], and the strength of this association was not reduced by inflammation (direct effect) [18]. Of note, the significant mediation effect of other inflammatory markers was repeatable in prevenient studies.

Thus, due to the rising prevalence and inadequate estimation of depression in PD patients, it is necessary to create early recognition and appropriate treatment of psychiatric symptoms and inflammatory response, avoiding severe somatic symptoms and enormous economic burden of PD patients.

### Limitations

However, as a retrospective analysis, there were some limitations in this study. First, the PD patients with history of malignant tumors were not included in this study, considering their short survival time and uncertain status of inflammation. It is reported that malignancy may cause “acute-phase response” and higher risk of infection [34]. In addition, acute infection complications with very high levels of CRP, such as peritonitis, or pulmonary infection, were also excluded while our research was focus on the low-grade inflammation. Perhaps further research designed to explore the relationship between these above factors are needed. Second, the dynamic changes in the level of depression and inflammation were not assessed during the follow-up period. Third, more biological markers representing stages of inflammation are also needed to extend the evidence of relationship between depression and inflammation. Finally, observational findings cannot determine causality, thus randomized trials for interventions about depression as well as inflammation are needed.

## Conclusion

The combined of depression and inflammation is associated with higher risk for all-cause mortality in PD patients. This suggests the need for more further studies on depression and low-grade inflammation and their potential relationship in these patients.

## Data Availability

The datasets used and/or analysed during the current study are available from the corresponding author on reasonable request.
